# GO/CdS Heterojunctions for Accelerated Photocatalytic Antibiotic Degradation

**DOI:** 10.3390/nano15191475

**Published:** 2025-09-26

**Authors:** Yutao Zhou, Kun Liu, Shuting Zhuang, Yunsong Mu

**Affiliations:** School of Chemistry and Life Resources, Renmin University of China, Beijing 100872, China; 2023101929@ruc.edu.cn (Y.Z.); lkun@ruc.edu.cn (K.L.); muyunsong@ruc.edu.cn (Y.M.)

**Keywords:** tetracycline, photodegradation, GO, CdS

## Abstract

The widespread detection of antibiotics in aquatic environments has raised significant concerns due to their potential risks to human health. Photocatalytic technology has emerged as an effective approach for antibiotic degradation, with cadmium sulfide (CdS) being a promising semiconductor photocatalyst. However, the practical application of CdS is limited by its tendency to aggregate, which reduces the number of accessible active sites and consequently lowers its photocatalytic degradation efficiency. In this study, a series of GO/CdS composites were synthesized via a two-step hydrothermal method for the efficient degradation of tetracycline (TC) antibiotics in aquatic solutions. Results showed that GO/CdS can effectively remove TC via photocatalytic degradation rather than adsorption. The optimized photocatalytic composite achieved a 95% degradation of TC (20 mg L^−1^) under 60 min of illumination. The corresponding rate constant (*k*) was 2.87 times higher than that of pristine CdS. After three cycles, the degradation rate still achieved 93%. Moreover, the composite exhibited a wide pH tolerance range from pH 2 to 10, with a removal rate of over 89%. Superoxide radicals (·O_2_^−^) were identified as the primary reactive species responsible for TC degradation, and three possible TC degradation pathways were proposed. This work extends the application of GO and offers a novel strategy for constructing GO-based composite materials, providing valuable insights into the mechanisms and pathways of antibiotic degradation.

## 1. Introduction

The widespread use of antibiotics in healthcare, livestock, and aquaculture industries [1,2] has led to the frequent detection of antibiotic residues in aquatic environments, posing significant threats to human health and ecosystems [3]. Due to the limited absorption capacity of antibiotics by both humans and livestock, a substantial proportion of administered antibiotics is excreted and subsequently discharged into surface waters [4]. Owing to their persistent nature and high stability, antibiotic residues can accumulate in aquatic environments. According to the World Health Organization, antibiotic contaminants have been widely detected in water bodies across the Western Pacific and Southeast Asia regions, with approximately 80–90% of untreated wastewater being discharged into water sources. Velpandian [5] reported the presence of 28 different types of antibiotic compounds in the surface waters of the Yamuna River.

Among various antibiotics, tetracyclines (TC) are broad-spectrum antibiotics widely used in recent decades, and their frequent detection in surface waters has raised significant concern. In China, high concentrations of TC antibiotics have been detected in multiple water systems. For instance, the highest concentration of TC reached 9500 ng L^−1^ in Wenyu River, followed by 8860 ng L^−1^ in Qing River, and 6800 ng L^−1^ in Tonghui River [6]. Similarly, Javid [7] detected TC concentrations ranging between 5.4 and 8.1 ng L^−1^ in groundwater samples from Tehran, Iran, with the highest level (9.3 ng L^−1^) recorded at the Yaftabad station. With the increasing global use of antibiotics, the accumulation of these compounds in surface waters continues to grow, posing serious threats to both human health and ecosystem integrity. However, conventional wastewater plants exhibit low efficacy in antibiotic removal [8]. Thus, the development of efficient antibiotic degradation technologies is urgently needed.

In recent years, various strategies for antibiotic removal have been explored, including membrane separation [9], photocatalysis [10], Fenton oxidation [11], and adsorption [12,13]. Among these strategies, semiconductor-based photocatalytic degradation has garnered significant attention due to its energy-efficient nature and high efficiency [14]. To date, many semiconductor photocatalysts have been developed for this purpose, including TiO_2_ [15], ZnO [16], WO_3_ [17], and CdS. When they are irradiated with sunlight, photogenerated holes and electrons react with water and dissolve oxygen to produce strongly oxidizing species capable of degrading antibiotics [18]. Therefore, semiconductor materials are regarded as potential candidates for pollutant degradation reactions [19].

Particularly, CdS (Eg = 2.4 eV) exhibits strong visible light absorption and rapid generation of electron-hole pairs. However, CdS still suffers from severe photocorrosion and rapid charge recombination, which limit its practical applications in antibiotic degradation [20]. Moreover, although the solubility of chromium is very low, it also needs to be tested through experiments in the actual process for risk considerations. Hence, effective modification of CdS is essential. Researchers have explored various strategies to mitigate these limitations, including morphological engineering [21], heterostructure construction [22], and integrative engineering. Among these strategies, integrating CdS with two-dimensional materials has emerged as a promising approach.

Graphene oxide (GO), when composited with CdS, can significantly enhance the photocatalytic degradation efficiency of pollutants due to its abundant oxygen-containing functional groups, large specific surface area, and excellent electron transport properties [23,24,25]. For example, Das [26] reported a ZnO-CdS-GO ternary heterostructure that exhibited a methyl orange degradation rate constant (1.6 × 10^−2^ min^−1^), 3.64 times higher than that of pure ZnO. Similarly, Yin [27] developed a CdS-rGO-ZnS composite via an emulsion solvothermal method, achieving 80.3% TC degradation within 10 min under visible light. Yang [28] employed carbon dots–CoO octahedrons as a photocatalyst and achieved up to 90% TC removal efficiency. However, current research on GO predominantly focuses on its composite applications for hydrogen or hydrogen peroxide production, while relatively limited attention has been paid to its potential in antibiotic degradation. The large specific surface area of GO nanosheets facilitates the adsorption of oxygen from both the solution and the atmosphere. This property not only promotes the separation of photogenerated electrons from the semiconductor, enabling more efficient participation in antibiotic degradation reactions [29], but also helps prevent the aggregation of CdS nanorods [30].

In this study, a carbon–semiconductor composite was fabricated by anchoring CdS nanorods onto GO using a two-step hydrothermal method for the photocatalytic degradation of TC antibiotics in aqueous solutions. The effects of varying GO/CdS ratios and pH conditions on degradation performance were systematically evaluated, and the dominant reactive species were identified through scavenger experiments. The material’s stability and selectivity toward tetracycline were confirmed through three recycling tests and experiments using real water samples. We further evaluated the solubility and release rate of heavy metal Cd^2+^ from the composite material over three consecutive cycles. In addition, photoluminescence (PL) and UV–visible spectroscopy (DRS) were employed to investigate the recombination rate of photogenerated electron-hole pairs in the composite. Based on the experimental results, plausible degradation pathways and mechanisms were proposed.

## 2. Materials and Methods

### 2.1. Chemicals and Reagents

All chemicals were of analytical grade and used without further purification. Deionized water was used for preparing all aqueous solutions. Cadmium nitrate tetrahydrate (Cd(NO_3_)_2_·4H_2_O), GO, thiourea (CH_4_N_2_S), ethylenediamine (EDA), and TC were purchased from Sigma-Aldrich (Saint Louis, MI, USA), Aladdin (Seattle, WA, USA), TCI Chemical (Shanghai, China), and Macklin company (Shanghai, China), respectively.

### 2.2. Preparation of Samples

#### 2.2.1. Synthesis of GO/CdS Nanocomposites

**Figure 1:**
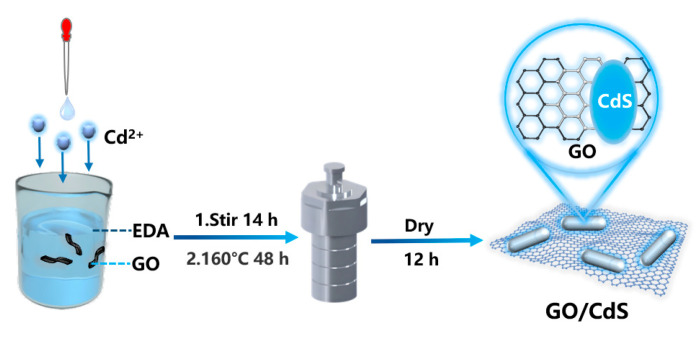
Overall steps diagram for synthesizing GO/CdS.

The GO/CdS nanocomposites were synthesized via a two-step hydrothermal method (Figure 1). Briefly, a certain amount of the as-prepared GO (percentage by weight of GO was 1 wt.%, 3 wt.% and 5 wt.%, respectively) was dispersed into 10 mL EDA (150 mmol) with ultrasonication for 1 h to form a homogeneous GO solution. After that, 2.31 g of Cd(NO_3_)_2_·4H_2_O (7.488 mmol) was dissolved in 15 mL of EDA (224 mmol) under continuous stirring. Subsequently, 1.715 g of thiourea (22.53 mmol) dissolved in 10 mL of EDA (150 mmol) was added dropwise to the above solution over 2 h under vigorous stirring. The resulting mixture was transferred into a 50 mL Teflon-lined stainless-steel autoclave and maintained at 160 °C for 48 h. After cooling naturally to room temperature, the product was collected by centrifugation, washed thoroughly with deionized water and ethanol several times, and finally dried at 65 °C for 12 h [31]. For clarity purposes, anchoring CdS onto GO prepared by using a different percentage by weight of GO are denoted as GCS-1, GCS-3, and GCS-5, respectively.

#### 2.2.2. Synthesis of CdS

In a typical synthesis, 25 mL of EDA (374 mmol) was added to 2.31 g of Cd(NO_3_)_2_·4H_2_O (7.488 mmol) under stirring. Subsequently, 10 mL of EDA (150 mmol) solution containing 1.715 g of thiourea (22.53 mmol) was slowly added dropwise to the mixture over 2 h under continuous stirring. The resulting mixture was transferred to a 50 mL Teflon-lined stainless-steel autoclave, sealed, and maintained at 160 °C for 48 h. After cooling naturally to room temperature, the product was washed several times with deionized water and ethanol, collected by centrifugation, and dried at 65 °C for 12 h.

### 2.3. Material Characterization

The morphological characteristics and elemental composition of the samples were characterized by scanning electron microscopy (SEM, FEI, Hillsboro, OR, USA) equipped with energy-dispersive X-ray spectroscopy (EDS, Oxford Instruments, Oxford, UK). X-ray diffraction (XRD, Bruker AXS, Karlsruhe, Germany) patterns were obtained using a Rigaku Ultima IV diffractometer with Cu Kα radiation (λ = 1.5406 Å), operating at 40 kV and 40 mA. The diffraction data were collected over the 2θ range of 10–80° with a scanning rate of 5° min^−1^. X-ray photoelectron spectroscopy (XPS) was performed using a PHI5300 spectrometer (ESCALAB, 250Xi, Thermo Scientific, Waltham, MA, USA) with an Al K (12.5 kV) X-ray source. The specific surface area and pore size distribution were analyzed by Brunauer–Emmett–Teller (BET) and Barret–Joyner–Halenda (BJH) methods, respectively. The UV–vis diffuse reflection spectra of the samples were examined by a UV–vis spectrophotometer (Hitachi U-4100, Shimadzu Corporation, Kyoto, Japan) in the range of 400–800 nm with BaSO_4_ as reference. The PL spectra of different samples were investigated by utilizing a fluorescence spectrophotometer (F-380, Hitachi, Tokyo, Japan)) equipped with a xenon lamp as the excitation source at room temperature. The excitation wavelength is 405 nm and the detection wavelength is 530 nm. Liquid Chromatograph Mass Spectrometry (LC-MS) was performed using a liquid chromatograph coupled to a TSQ Quantis (Thermo Scientific, Waltham, MA, USA) equipped with a C18 column (100 mm × 2.1 mm, 1.7 μm).

### 2.4. Photocatalytic Degradation Experiments

The photocatalytic activity of the GO/CdS nanocomposites was evaluated using TC as the target pollutant. A 350 W xenon lamp (BBZM-I, 300 W, Chenhua, China) equipped with a UV-cutoff filter (λ > 420 nm) served as the visible light source. Prior to illumination, 10 mg of the photocatalyst was dispersed in 100 mL of TC aqueous solution (20 mg L^−1^) and magnetically stirred in the dark for 30 min to achieve adsorption–desorption equilibrium.

During the whole stirring process, 0.8 μL aliquots were collected at 10 min intervals. Each sample was immediately filtered through a 0.22 μm membrane filter to remove photocatalytic particles. The residual TC concentration was analyzed by high-performance liquid chromatography (HPLC). The column temperature was 30 °C, the detection wavelength was 357 nm, the flow rate was 0.8 mL min^−1^ and the injection volume was 20 μL. The aqueous phase A was 0.01 mol L^−1^ oxalic acid aqueous solution, and the mobile phase B was chromatographic grade methanol solution. The ratio of A to B was 1:1.

The degradation efficiency was calculated according to the following equation:

(1) $$Degradation\;efficiency\;=\;\left[\frac{C_{i}-C_{f}}{C_{i}}\right]\;\times \;100\%$$

where $C_{i}$ is the initial concentration of TC, while $C_{f}$ represents the concentration of TC after a degradation period of t.

The effectiveness of the photocatalytic treatments for TC was evaluated by conducting the total organic carbon (TOC), which was determined by a TOC analyzer (Shimadzu, Kyoto, Japan): 10 mg GCS-3 added to 100 mL TC solution (20 mg L^−1^) and magnetically stirred in the dark for 30 min to achieve adsorption–desorption equilibrium. Then, the solution was exposed to visible light for 60 min. After 60 min illumination, the resulting sample was filtered through a 0.22 μm membrane filter to remove photocatalytic particles. Then, the sample was subjected to the standard protocols of TOC. The percentage of TOC reduction was calculated using the following equation:

(2) $$TOC\;=\;\left[\frac{TOC_{i}-TOC_{f}}{TOC_{i}}\right]\;\times \;100\%$$

where $TOC_{i}$ and $TOC_{f}$ represents the initial and final values of TOC, respectively.

Radical scavenging experiments were carried out in order to deeply clarify photocatalytic mechanisms: Benzoquinone (BQ, ·O_2_^−^ scavenger), ethylenediaminetetraacetic acid disodium salt (EDTA-2Na, h^+^ scavenger), ethanol (e^−^ scavenger), and isopropanol (IPA, OH^−^ scavenger) were applied to investigate the possible dominant reactive species in the TC degradation process. The photocatalytic suspensions were quantitatively recovered through vacuum filtration using a 0.22 μm membrane filter. The collected photocatalysts were subsequently dried at 65 °C for 12 h to ensure cycling experiments.

The cycling experiments were performed under identical conditions to ensure comparability: 10 mg GCS-3 was added in 100 mL TC solution (20 mg L^−1^) with 30 min dark stirring, followed by 60 min visible light illumination. Sample collection and analysis followed the same procedure as described above.

## 3. Results and Discussion

### 3.1. Characterization Results

**Figure 2:**
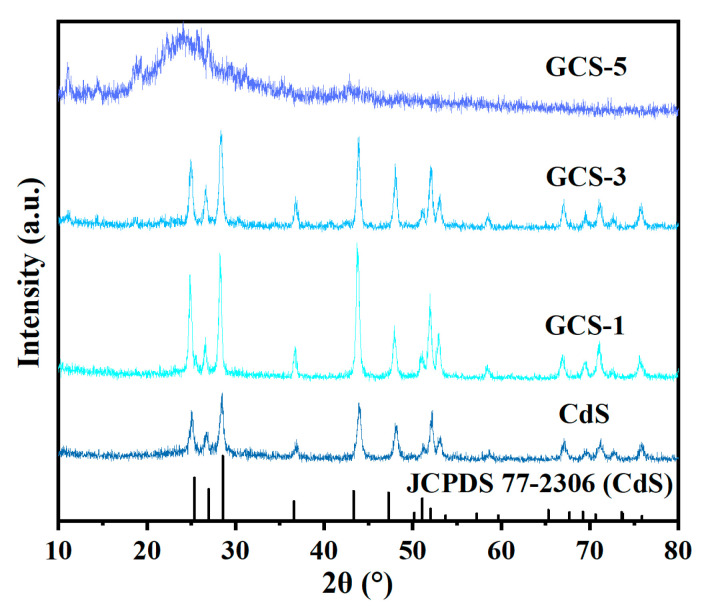
XRD pattern of the prepared photocatalysts.

Figure 2 presents the XRD patterns of as-synthesized samples. Diffraction peaks for pure CdS and GO/CdS composites (GCS-1 and GCS-3) could be marked as CdS with the hexagonal wurtzite structure (JCPDS 77-2306). The characteristic diffraction peaks at 2θ = 24.8°, 26.5°, 28.2°, 36.6°, 43.7°, 47.8°, and 51.8° corresponded to the (100), (002), (101), (102), (110), (103), and (112) crystal planes of hexagonal CdS, respectively [32]. These results confirmed that the load of GO did not change the crystalline structure of CdS. Moreover, when GO was incorporated, the characteristic of CdS peaks exhibited increased intensity and a slight shift toward lower 2θ values. This observation suggested an expansion of the interplanar spacing and improved crystallinity of CdS within the composite material. Interestingly, the GCS-5 composite exhibited distinct differences in its XRD pattern. A characteristic GO peak appeared at 2θ = 11.0° [33], this phenomenon suggested that excessive GO loading leads to agglomeration, which effectively encapsulated the CdS crystallites within the GO layers.

**Figure 3:**
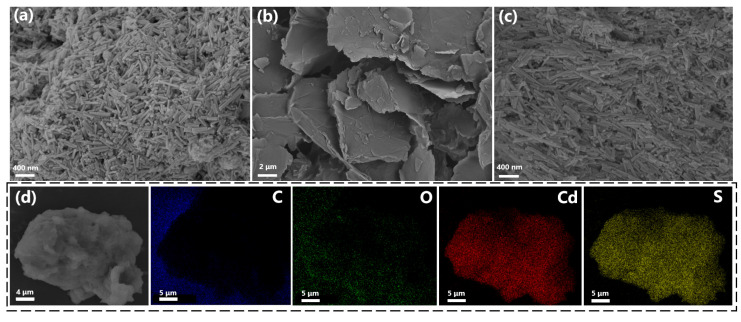
SEM and EDS images of composite materials (**a**) GCS-3 (**b**) GCS-5 (**c**) CdS (**d**) EDS image of GCS-3.

SEM was employed to characterize the morphology of the synthesized composites. Figure 3a–c displayed the SEM images of GCS-3, GCS-5, and pure CdS, respectively. As depicted in Figure 3a,c, both GCS-3 and pure CdS exhibited uniform nanorod morphologies with an average length of approximately 400 nm and diameter of 50 nm [34]. This observation correlated well with the preferred crystalline orientation revealed by the XRD analysis. However, the GCS-5 composite (Figure 3b) showed complete morphological transformation, where the nanorod structure was replaced by sheet-like features resembling pristine GO [35]. This transition suggested that excessive GO loading leads to complete encapsulation of CdS nanorods by GO sheets, obscuring their visibility in SEM and aligning with the appearance of a GO characteristic peak in XRD.

The elemental composition of GCS-3 composite was further investigated using energy dispersive spectrometer (EDS) and elemental mapping (Figure 3d). The results confirmed the homogeneous distribution of C, O, Cd, and S throughout the sample [36]. The spatial distribution of Cd and S exhibited a high degree of overlap, corroborating the structural integrity of the CdS nanorods. Conversely, C and O demonstrated a pronounced enrichment at the edges of the CdS nanorods, forming a clear phase interface between the CdS region and the GO component. These results provided strong support for the successful integration of CdS nanorods with GO nanosheets in the composite.

**Figure 4:**
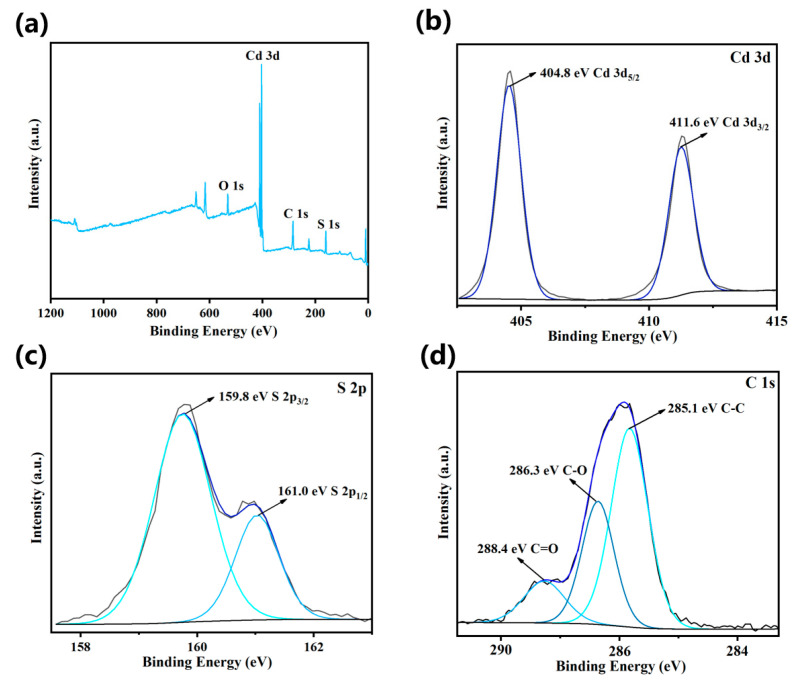
(**a**) Survey spectrum of GCS-3 nanocomposite and deconvoluted XPS spectra of (**b**) Cd 3d, (**c**) S 2p, (**d**) C 1s.

To investigate the elemental composition and chemical states of the GCS-3 composite photocatalyst, XPS analysis was conducted. The survey spectrum of the composite (Figure 4a) clearly indicated the presence of C, O, Cd, and S, suggesting no significant impurities were introduced during the synthesis process, which were consistent with the EDS results. High-resolution XPS spectra of Cd 3d, S 2p, and C 1s are presented in Figure 4b–d, respectively. In the Cd 3d spectrum (Figure 4b), two sharp and symmetric peaks were observed at binding energies of 404.8 eV and 411.6 eV, corresponding to Cd 3d_5/2_ and Cd 3d_3/2_, respectively. These values were characteristic of Cd^2+^ in CdS [37]. The S 2p spectrum (Figure 4c) exhibited two peaks at 159.8 eV and 161.0 eV, which can be assigned to S 2p_3/2_ and S 2p_1/2_, confirming the presence of S^2−^ [38]. Furthermore, the C 1s spectrum (Figure 4d) displayed a major peak at 285.1 eV, attributed to the C-C bond. Additional peaks observed at 286.3 eV and 288.4 eV were associated with C–O (hydroxyl) and C=O (carbonyl) groups, which were consistent with the characteristic functional groups of GO. These results confirmed the successful formation of the GCS-3 composite with the expected chemical environment.

**Figure 5:**
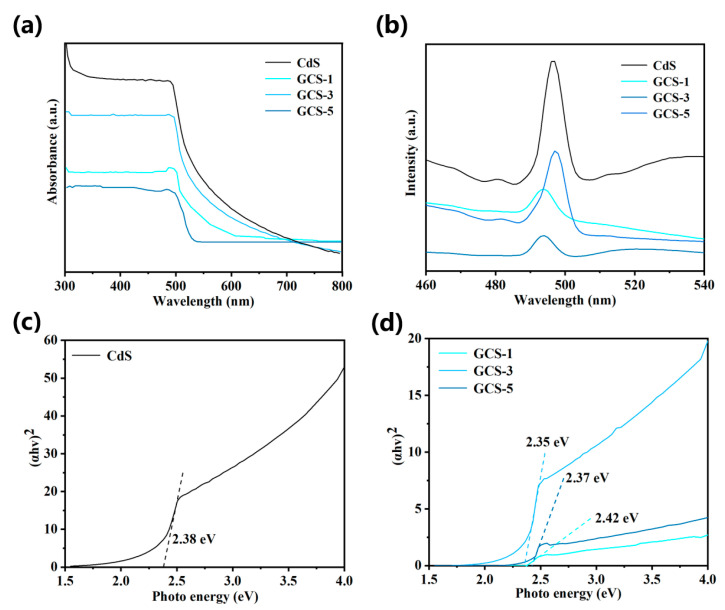
(**a**) DRS of composite materials (**b**) PL images of composite materials (**c**) Tauc plot of CdS (**d**) Tauc plot of composite materials.

The light absorption capability of the composite materials within the visible range is critically important, and is essential for efficient photocatalytic reactions. DRS was employed to characterize the optical absorption properties of CdS, GCS-1, GCS-2, and GCS-3 composites over the wavelength range of 300–800 nm. As shown in Figure 5a, pure CdS exhibited a typical absorption profile of hexagonal wurtzite-type CdS, with an absorption edge around 551 nm [39], which was attributed to the intrinsic bandgap transition from the valence band to the conduction band [40]. After modification with GO, the absorption intensity increased significantly from GCS-1 to GCS-3 but decreased sharply beyond GCS-3 to GCS-5. This suggested that GCS-3 possesses the strongest light-harvesting ability among the composites, which was consistent with its superior performance in the photocatalytic degradation of TC. The decline in absorption of GCS-5 may be due to excessive coverage of GO, which reduced light penetration and absorption. Nevertheless, all composite samples demonstrated higher degradation efficiency than pure CdS, confirming that the incorporation of GO enhanced the overall light utilization efficiency.

The PL is related to the recombination of electron-hole pairs; lower PL intensity indicates suppressed charge recombination, while higher intensity suggests more rapid recombination [41]. Figure 5b presents the room-temperature PL spectra of CdS, GCS-1, GCS-3, and GCS-5. It was evident that all composites exhibited lower emission intensities than pure CdS, indicating reduced charge recombination after coupling with GO. Among them, GCS-3 showed the lowest PL intensity, implying the most effective separation of photogenerated carriers, which correlated well with its highest photocatalytic activity. The Tauc plots derived from (αhν)^2^ versus photon energy (hν) are shown in Figure 5c,d. The bandgap of CdS, GCS-1, GCS-3, and GCS-5 were 2.38 eV, 2.42 eV, 2.35 eV, and 2.37 eV, respectively. The redshift in the absorption edge and reduced bandgap for GCS-3 and GCS-5 may be attributed to interactions between CdS nanoparticles and graphene sheets [42]. The increased bandgap of GCS-1 was consistent with previous reports [43], and may result from quantum confinement effects due to the smaller particle size of CdS.

### 3.2. Photocatalytic Degradation Performance

**Figure 6:**
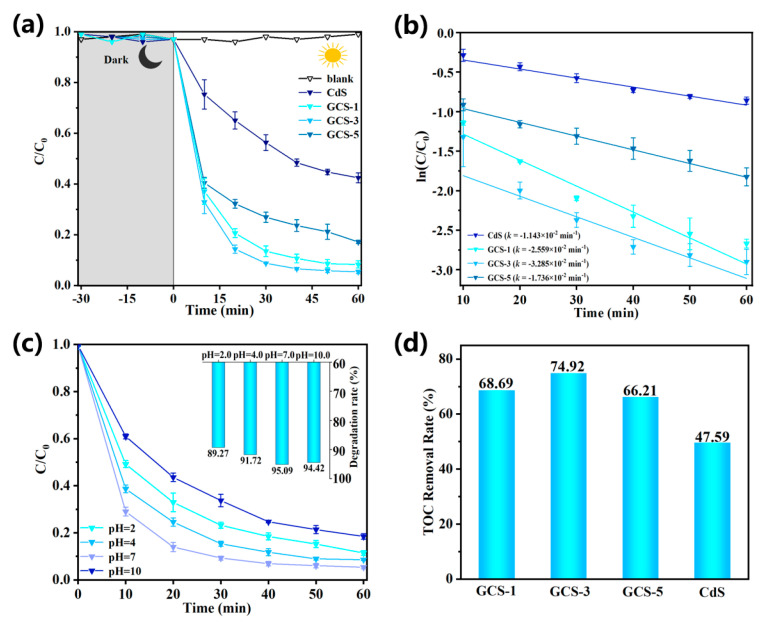
(**a**) Photocatalytic degradation of GO/CdS composite materials. (**b**) Pseudo-first-order kinetic diagram of composite materials. (**c**) Effect of different pH on GCS-3 photocatalytic degradation (**d**) TOC removal ratio of GO/CdS composite materials.

The assessment of photocatalytic degradation efficiency is a crucial method for evaluating the degradation characteristics of semiconductor materials and their efficacy in photocatalytic applications. As shown in Figure 6a, the concentration of TC remained relatively stable across all samples after 30 min of dark adsorption, indicating that the contribution of adsorption to the overall degradation process was negligible. This aligned with the findings reported in previous studies, confirming the consistency of the proposed degradation mechanisms within the existing body of research [44]. Furthermore, the blank solution exhibited minimal self-photodegradation after 60 min of visible light illumination in the absence of any photocatalyst, verifying the absence of spontaneous photolysis within the system. The GCS-3 composite demonstrated the highest photocatalytic degradation activity and achieved a degradation efficiency of over 95% after 60 min of visible light irradiation. The GCS-1 composite exhibited a slightly lower degradation efficiency of approximately 91% under the same conditions. It was noteworthy that increasing the GO weight ratio to 5 wt.% led to a downward trend in the degradation efficiency to approximately 82% after 60 min. This reduction may be attributed to the aggregation of excess GO, leading to a diminished surface area of the GO/CdS composite [45]. This could cause a screening effect, hindering the separation of photogenerated electron-hole pairs in CdS. Despite the decline at higher GO loadings, all GO/CdS composites showed significantly improved photocatalytic performance compared to pure CdS, which showed only about 58% degradation efficiency after 60 min. This underscored the beneficial effect of GO integration in enhancing the photocatalytic performance of CdS and demonstrated a superior TC degradation efficiency compared to previously reported GO/CdS composites.

To quantitatively evaluate the photocatalytic performance and probe the degradation kinetics of the synthesized materials, TC degradation process was analyzed using a pseudo-first-order kinetic model. The kinetic fitting results, presented in Figure 6b, demonstrated that the photocatalytic degradation of TC by all synthesized catalysts conformed well to pseudo-first-order reaction kinetics [46]. Notably, the GCS-3 composite exhibited the highest apparent rate constant for TC degradation (*k* = −3.285 × 10^−2^ min^−1^), consistent with the previously observed superior photocatalytic activity. The apparent rate constants for the GCS-1 and GCS-5 composites were determined to be *k* = −2.599 × 10^−2^ min^−1^ and *k* = −1.736 × 10^−2^ min^−1^, respectively. Overall, the comparative analysis revealed that all GO/CdS composites showed greatly improved photocatalytic activity compared to pure CdS nanoparticles (*k* = −1.143 × 10^−2^ min^−1^). This enhancement could be owed to the incorporation of GO that effectively facilitated the separation and transportation of photogenerated charge carriers, leading to a substantial enhancement in the material’s photo-quantum efficiency [47].

The solution pH is a critical parameter influencing the degradation process by affecting the surface charge of the photocatalytic material, the degradation mechanism and the overall reaction rate [2]. Figure 6c illustrates the impact of varying pH values on the photocatalytic degradation rate of TC by the GCS-3 composite. When pH increases from 2.0 to 10.0, there is no obvious impact on photocatalytic degradation efficiency. Meanwhile, the composite exhibited optimal photocatalytic performance under neutral conditions (pH = 7.0), achieving 95.09%. This showed that under neutral pH, the photocatalyst achieves maximum degradation efficiency. Moreover, under extreme acidic (pH = 2.0) and alkaline (pH = 10.0) conditions, the photocatalytic activity degradation efficiency still retained 89.27% and 94.42%, respectively. This result demonstrated remarkable pH tolerance and versatility for practical wastewater treatment applications of the composite [48]. The change in electrostatic interactions between the TC molecules and catalyst surface may play a crucial rate-determining role.

To further evaluate the degradation ability from as-prepared samples, the mineralization of TC was measured by TOC testing. As seen in Figure 6d, the TOC removal rate of pure CdS only achieved 47.59%. Following the loading of GO, the TOC removal ratio of all GO/CdS composites surpassed that of pure CdS. In particular, the GCS-3 removal rate of TOC reached up to 74.92% within 60 min, which was consistent with previous research findings. Additionally, the GCS-1 and GCS-5 samples exhibited TOC removal rates of 68.69% and 66.21%, respectively. These results demonstrated that GO incorporation enhanced the mineralization efficiency of CdS, thereby enabling more effective degradation of TC in aqueous solutions.

**Figure 7:**
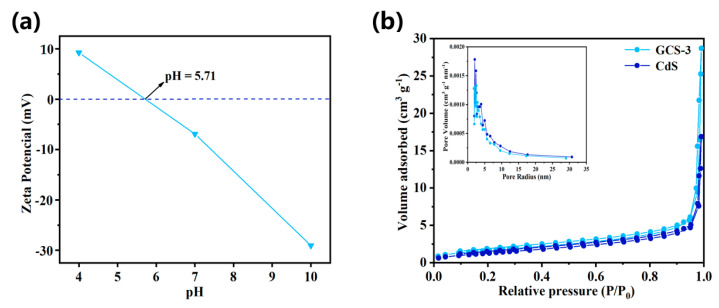
(**a**) Zeta potential of GCS-3 in different pH. (**b**) N_2_ adsorption–desorption isotherms of CdS and GCS-3 nanocomposites, inserts are the pore size distribution curves.

The zeta potential of the GCS-3 is shown in Figure 7a. The zeta potential gradually decreased with increasing pH, reaching the point of zero charge at pH = 5.71. Below this pH, the surface of GCS-3 was positively charged, while pH > 5.71, it became negatively charged. These results indicated that at pH < 5.71, TC dissociated into the cationic form TCH_3_^+^ [49]. Electrostatic repulsion between the positively charged GCS-3 surface and TCH_3_^+^ lead to reduced adsorption capacity. Within the pH range of 5.71–7.0, TC existed predominantly as the zwitterionic species TCH_2_^+^ [50]. In this range, the negatively charged GCS-3 surface facilitated adsorption through hydrophobic interactions and electrostatic attraction, resulting in optimal adsorption performance. Conversely, at pH > 7.0, TC primarily formed anionic species (TCH^−^ and TC^2−^), and electrostatic repulsion with the negatively charged adsorbent again reduced adsorption efficiency. Heterogeneous photocatalytic reactions were strongly influenced by the structural properties of the photocatalyst [51]. Therefore, N_2_ adsorption–desorption analysis was conducted. The isotherms and pore size distribution were presented in Figure 7b. Both CdS and the GCS-3 composite exhibited type IV isotherms with H3-type hysteresis loops [52,53], which were characteristic of mesoporous materials. The BET surface area, pore volume, and average pore size of CdS and GCS-3 were summarized in Table 1. Compared to 5 m^2^ g^−1^ for pure CdS, the specific surface area of GCS-3 was measured to be 6 m^2^ g^−1^. The increased surface area of the composite likely contributed positively to its enhanced photocatalytic performance. Additionally, the average pore size of GCS-3 was notably larger than that of CdS. These results further confirmed the successful modification and presence of abundant active sites on the composite material.

Table 2 lists some related works on the performance of various CdS-based nanocomposite materials in photocatalytic reactions for TC degradation. Notably, the degradation efficiency using GO/CdS as the photocatalyst exhibited excellent removal ability, achieving a removal rate of up to 95% after only 60 min, which is higher than other methods. In addition, introducing GO into CdS has been widely used to enhance photocatalytic activity. For example, Guan [54] synthesized Z-scheme photocatalysts by modifying GO with AgBr nanoparticles and coupling it with Bi_2_WO_6_. Under visible light irradiation, these catalysts demonstrated a maximum degradation efficiency of up to 84%. Firouzi [55] employed CdS-TNs/rGO to facilitate the photocatalytic degradation of tetracycline hydrochloride, achieving degradation efficiency of 84% after 180 min of illumination.

### 3.3. Photocatalytic Degradation Mechanism

#### 3.3.1. Active Species Identification

**Figure 8:**
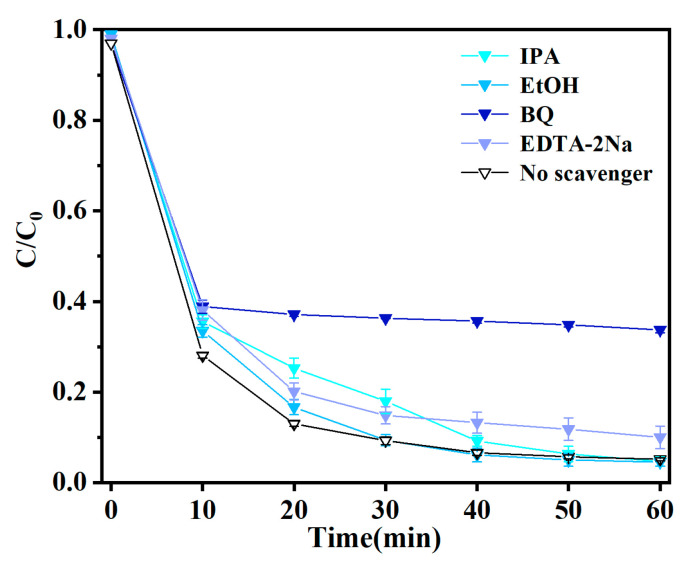
Experimental diagram of active species capture of GCS-3.

To identify the predominant reactive species involved in the photocatalytic degradation of TC by the GCS-3 composite, radical scavenging experiments were conducted [64]. BQ, EDTA-2Na, ethanol, and IPA were employed to investigate the possible active species in the TC degradation process (Figure 8). The experimental data revealed that the introduction of BQ significantly suppressed the photocatalytic degradation, indicating that ·O_2_^−^ play a dominant influence in the degradation process. The predominant role of ·O_2_^−^ aligned with findings reported in previous studies of similar systems [65]. In contrast, the introduction of EDTA-2Na, ethanol, and IPA resulted in only a marginal reduction in the degradation efficiency. This finding further corroborated that ·O_2_^−^ was the primary reactive species involved in the photocatalytic reaction.

#### 3.3.2. Proposed Degradation Pathways

**Figure 9:**
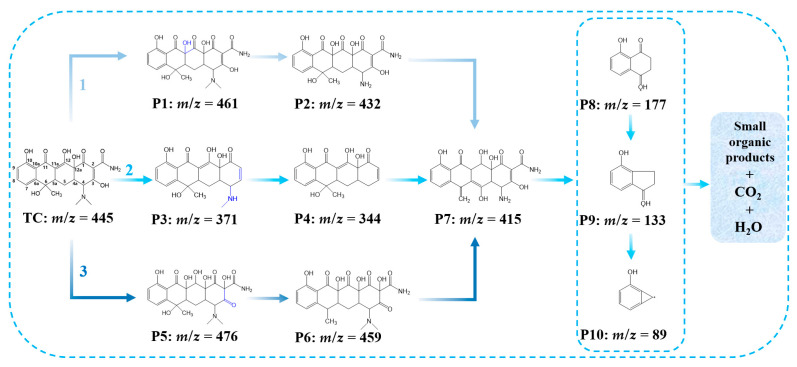
Possible degradation pathways diagram of composite materials.

To elucidate the degradation pathways of TC in the GO/CdS system, we employed LC-MS to identify potential intermediate products. The LC-MS analysis revealed the presence of several intermediates, exhibiting *m*/*z* values of 476, 461, 432, 415, 371, 344, 177, 133, 89, and, additionally, the parent TC molecule (assigned *m*/*z* = 445). Based on the identified intermediates and predicted reaction sites, three plausible TC degradation pathways were proposed, as shown in Figure 9.

In pathway 1, the C11a-C12 double bond, being the most reactive site [66], underwent ·OH^−^ attack to form intermediate P1 (*m*/*z* = 461) through hydroxylation, generating a resonance-stabilized carbon-centered radical [67,68]. Subsequent radical-mediated N-demethylation removed two methyl groups, yielding intermediate P2 (*m*/*z* = 432) [69]. Further dehydration and hydroxylation produced intermediate P7 (*m*/*z* = 415), ultimately leading to ring-opening and formation of small molecular compounds.

In pathway 2, degradation initiated at the C4-dimethylamino group via N-dealkylation. Intermediate P3 (*m*/*z* = 371) formed through sequential loss of N-methyl, hydroxyl, and amide groups, consistent with previous reports on TC oxidation by ferrate [70].

In pathway 3, simultaneous attacks on both C11a-C12 and C2-C3 double bonds (the second most vulnerable site) by hydroxyl radicals generated intermediate P5 (*m*/*z* = 476). The predominance of OH^−^-mediated reactions was attributed to their abundance and strong oxidative capacity [71]. This pathway proceeded through 1,3-dipolar cycloaddition at the double bonds followed by ·OH^−^-induced rearrangement at C12 and C3 positions.

Notably, low molecular weight fragment was also detected, indicating complete mineralization of some TC molecules. The coexistence of multiple pathways demonstrated the comprehensive degradation capability of the GO/CdS system.

Based on the experimental outcomes, we proposed the following photocatalytic mechanisms:

1. Under visible light irradiation, CdS underwent intrinsic bandgap excitation, generating electron-hole pairs through promotion of valence band electrons to the conduction band;

2. The significant Fermi level difference between GO and CdS drove rapid electron transfer from the CdS conduction band to the GO plane;

3. The accumulated electrons on the GO surface participated in multi-electron reduction of adsorbed oxygen:


(3) $$O_{2}+e^{-}\to \cdot O_{2}^{-}$$

These ·O_2_^−^ then initiated nucleophilic attacks on the conjugated system of TC molecules, leading to ring-opening reactions and subsequent mineralization processes.

### 3.4. Photocatalytic Performance in Real Water Systems and Recyclability

To evaluate the potential of the GO/CdS composite for pollutant degradation in real aquatic environments, the photocatalytic activity of the GCS-3 composite was assessed for TC degradation using natural lake water as the aqueous matrix. The lake water was collected from Fangzhou Lake (Beijing, China). Table 3 showed the physicochemical properties of the real water samples.

**Figure 10:**
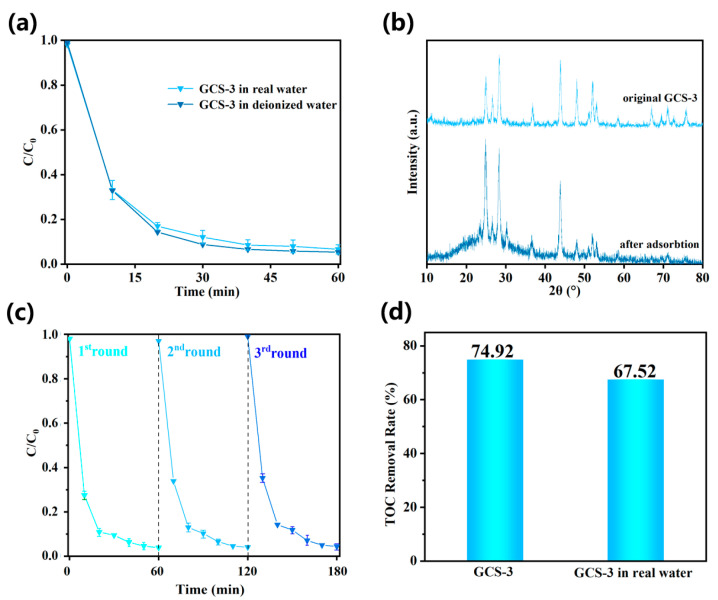
(**a**) Degradation experiment of GCS-3 in real water. (**b**) XRD pattern of GCS-3 after degradation experiment in real water. (**c**) Experimental diagram of cyclic degradation of GCS-3 composite material. (**d**) TOC removal ratio of GCS-3 in real water and deionized water.

As shown in Figure 10a, the GCS-3 composite exhibited a TC degradation efficiency exceeding 92% in natural lake water, which was comparable to that in deionized water. This indicated that the composite maintained high photocatalytic performance under more complex environmental conditions and suggested feasibility for real water treatment applications [72]. Figure 10b presents the XRD patterns of the GCS-3 composite after photocatalytic degradation of TC in natural lake water. Notably, the intensity of the (100), (101), and (110) diffraction peaks, characteristic of CdS, increased, while the intensity of the (002), (103), and (112) peaks decreased. This observation may be owed to the existence of various ions in the lake water, which could adsorb onto the CdS surface, thereby altering the electronic structure of the CdS component and influencing the diffraction peak intensities. Furthermore, these ions may affect the dissolution–recrystallization process of CdS, potentially promoting crystal growth and enhancing the overall crystallinity. Despite these changes in peak intensities, the overall XRD pattern of GCS-3 remained largely similar to that of the pristine material, suggesting that the composite maintained its structural integrity and exhibited reasonable stability under real operational conditions. This stability further verified its potential for practical implementation in environmental remediation technologies.

The stability of photocatalyst is a critical index for evaluating its potential for practical application [73,74]. To assess the photocatalytic stability of the GCS-3 composite, cyclic photodegradation experiments were performed on TC solutions. As shown in Figure 10c, the photocatalytic activity of the GCS-3 composite exhibited only a slight decrease after three successive photodegradation cycles. Specifically, the degradation efficiency slightly decreased from 95% in the first cycle to 92% in the third cycle. These results indicated that the GCS-3 composite possessed good photostability and demonstrated potential for the repeated degradation of tetracycline in environmental applications. To evaluate the low environmental toxicity of the composite material, atomic absorption spectroscopy (AAS) was employed to determine the concentration of Cd^2+^ ions leached into the solution after three cycles of tetracycline photocatalytic degradation. The results indicated that the released Cd^2+^ concentrations were within the range of 1.816 × 10^−8^ g L^−1^ and 2.508 × 10^−8^ g L^−1^, which were below the permissible limits for cadmium in drinking water and groundwater standards. These findings demonstrated the minimal toxicity of the composite and suggested its potential suitability for antibiotic degradation in real aquatic environments without posing substantial risks to human health or ecosystems. Figure 10d shows the comparison of the TOC removal ratio between deionized water and real water samples following photocatalytic degradation using GCS-3. The results indicated that in natural water, the TOC removal rate of GCS-3 decreased to 67.52%. However, this value remained higher than that achieved with pure CdS under identical conditions. This reduction in removal efficiency was likely attributable to interference from various ions presenting in the natural water matrix, which may hinder the photocatalytic performance of the composite material.

## 4. Conclusions

In this study, GO/CdS composites with varying percentage by weight of GO (1 wt.%, 3 wt.%, and 5 wt.% GO) were synthesized via a two-step hydrothermal method. Characterization by XRD, SEM, and EDS confirmed the successful loading of CdS nanorods onto the GO sheets. Photocatalytic activity tests confirmed that the degradation of TC by the composites occurred primarily through photocatalytic processes with minimal contribution from adsorption processes. The GCS-3 composite exhibited the highest photocatalytic efficiency, achieving over 95% TC degradation within 60 min. Notably, all composites demonstrated TC degradation efficiencies exceeding 80%, significantly outperforming pristine CdS (58%). This enhanced performance was attributed to the rapid electron transport facilitated by GO, which effectively suppressed the recombination of photogenerated electron-hole pairs in CdS. The pH dependence studies showed that the GCS-3 composite exhibited optimal degradation activity (95%) under neutral conditions (pH = 7.0) and wide pH tolerance. Radical scavenging experiments identified ·O_2_^−^ as the primary reactive species in the degradation process. After three cycles and in real water, the GCS-3 composite maintained over 90% of TC degradation, demonstrating its efficacy in complex environmental matrices and suitability for practical applications. Based on the above results, three possible degradation pathways and mechanisms for TC and prominent intermediate products were proposed.

## Figures and Tables

**Table 1:** The BET surface area and pore characteristics of the CdS and GCS-3.

Photocatalyst	BET Surface (m^2^ g^−1^)	Pore Volume (cm^3^ g^−1^)	Pore Diameter (nm)
CdS	5	0.026	20.40
GCS-3	6	0.043	28.61

**Table 2:** Comparison of photocatalytic activity between GO/CdS and reported photocatalysts for TC degradation.

Photocatalysts	Photocatalytic Conditions			Degradation Rate (%)	Ref.
	C_0_ (mg L^−1^)	Dosage of Catalysts (mg)	Time (min)		
GO/CdS	20	10	60	95	This work
CdS/CdSnO_3_	30	20	60	92	[56]
CMG-10	10	5	60	91	[57]
KPCN/GO/ZnFe_2_O_4_	36	60	60	87	[47]
GZF-25	10	5	60	87	[58]
AgBr/GO/Bi_2_WO_6_	20	40	60	84	[54]
CdS-TNs/rGO	15	75	180	84	[55]
GTPCG-20	20	50	120	82	[59]
rGO–CdS/ZnS	10	50	60	80	[27]
Ag_3_PO_4_/GO	10	25	30	75	[60]
rGO@PANI-Ca:Mg	10	2	60	71	[61]
2 wt% BWO-GO	10	100	60	63	[62]
N–ZnO/CdS/GO	20	50	60	4	[63]

**Table 3:** The physicochemical properties of the real water samples.

The Physicochemical Properties of Fangzhou Lake (Beijing, China)	
pH	7.1
COD (mg L^−1^)	17.3
T (°C)	25.0
TC concentration (mg L^−1^)	20.0
Color	Clear and transparent with suspended particles

## Data Availability

The data presented in this study are available on request from the corresponding author.

## References

[1] Baaloudj O., Assadi I., Nasrallah N., Jery A.E., Khezami L., Assadi A.A. (2021). Simultaneous removal of antibiotics and inactivation of antibiotic-resistant bacteria by photocatalysis: A review. J. Water Process Eng..

[2] Li D.W., Yu S.Y., Geng H.J., Zhou W., Mu D.D., Liu S.T. (2023). The (002) exposing facets of WO_3_ boosting photocatalytic degradation of nitrobenzene. Appl. Surf. Sci..

[3] Galloni M.G., Falletta E., Mahdi M., Cerrato G., Giordana A., Boffito D.C., Bianchi C.L. (2024). An Innovative Sunlight-Driven Device for Photocatalytic Drugs Degradation: From laboratory- to real-Scale Application. A First Step Toward Vulnerable Communities. Adv. Sustain. Syst..

[4] Han J., He S.S., Lichtfouse E. (2023). Waves of pharmaceutical waste. Environ. Chem. Lett..

[5] Velpandian T., Halder N., Nath M., Das U., Moksha L., Gowtham L., Batta S.P. (2018). Un-segregated waste disposal: An alarming threat of antimicrobials in surface and ground water sources in Delhi. Environ. Sci. Pollut. Res..

[6] Xu Y., Guo C.S., Luo Y., Lv J.P., Zhang Y., Lin H.X., Wang L., Xu J. (2016). Occurrence and distribution of antibiotics, antibiotic resistance genes in the urban rivers in Beijing, China. Environ. Pollut..

[7] Javid A., Mesdaghinia A., Nasseri S., Mahvi A.H., Alimohammadi M., Gharibi H. (2016). Assessment of tetracycline contamination in surface and groundwater resources proximal to animal farming houses in Tehran, Iran. J. Environ. Health Sci. Eng..

[8] Liu S.A., Zhao X.R., Sun H.Y., Li R.P., Fang Y.F., Huang Y.P. (2013). The degradation of tetracycline in a photo-electro-Fenton system. Chem. Eng. J..

[9] Cheng X.J., Liao J.H., Xue Y., Lin Q.Q., Yang Z.M., Yan G.L., Zeng G.Y., Sengupta A. (2022). Ultrahigh-flux and self-cleaning composite membrane based on BiOCl-PPy modified MXene nanosheets for contaminants removal from wastewater. J. Membr. Sci..

[10] Bembibre A., Benamara M., Hjiri M., Gómez E., Alamri H.R., Dhahri R., Serrà A. (2022). Visible-light driven sonophotocatalytic removal of tetracycline using Ca-doped ZnO nanoparticles. Chem. Eng. J..

[11] Zhang N.Q., Chen J.Y., Fang Z.Q., Tsang E.P. (2019). Ceria accelerated nanoscale zerovalent iron assisted heterogenous Fenton oxidation of tetracycline. Chem. Eng. J..

[12] Zhuang S.T., Zhu X., Wang J.L. (2018). Kinetic, equilibrium, and thermodynamic performance of sulfonamides adsorption onto graphene. Environ. Sci. Pollut. Res..

[13] Zhuang S.T., Wang J.L. (2021). Adsorptive removal of pharmaceutical pollutants by defective metal organic framework UiO-66: Insight into the contribution of defects. Chemosphere.

[14] Wang D.B., Jia F.Y., Wang H., Chen F., Fang Y., Dong W.B., Zeng G.M., Li X.M., Yang Q., Yuan X.Z. (2018). Simultaneously efficient adsorption and photocatalytic degradation of tetracycline by Fe-based MOFs. J. Colloid Interface Sci..

[15] Xu D., Ma H.L. (2021). Degradation of rhodamine B in water by ultrasound-assisted TiO_2_ photocatalysis. J. Clean. Prod..

[16] Fatima H., Azhar M.R., Khiadani M., Zhong Y.J., Wang W., Su C., Shao Z.P. (2022). Prussian blue-conjugated ZnO nanoparticles for near-infrared light-responsive photocatalysis. Mater. Today Energy.

[17] Li J.J., Li Y., Chen M.H., Tang X., Zhu N.W., Li W.X., Mei Q., Yue S.J., Tang Y.P., Wang Q.Z. (2023). Construction of polynary systems by coupling Cd/CdS with magnetic recyclable CuO/Fe_2_O_3_/CuFe_2_O_4_ nanocomposite for enhancing photo-Fenton degradation of antibiotics. J. Environ. Chem. Eng..

[18] Low J.X., Yu J.G., Jaroniec M., Wageh S., Al-Ghamdi A.A. (2017). Heterojunction Photocatalysts. Adv. Mater..

[19] Zeng F.G., Chen H.H., Mei Y.C., Ye L.B., Zhuang S.T., Pu N., Wang L.M. (2024). Performance and mechanism of sulfonamide-antibiotic adsorption by Ti_3_C_2_ MXene. New J. Chem..

[20] Chava R.K., Son N., Kang M. (2022). Bismuth quantum dots anchored one-dimensional CdS as plasmonic photocatalyst for pharmaceutical tetracycline hydrochloride pollutant degradation. Chemosphere.

[21] Zhou P.P., Le Z.G., Xie Y., Fang J., Xu J.W. (2017). Studies on facile synthesis and properties of mesoporous CdS/TiO_2_ composite for photocatalysis applications. J. Alloys Compd..

[22] Dou M.Y., Han S.R., Du X.X., Pang D.H., Li L.L. (2020). Well-defined FeP/CdS heterostructure construction with the assistance of amine for the efficient H_2_ evolution under visible light irradiation. Int. J. Hydrogen Energy.

[23] Lv N., Li Y.Y., Huang Z.L., Li T., Ye S.Y., Dionysiou D.D., Song X.L. (2019). Synthesis of GO/TiO_2_/Bi_2_WO_6_ nanocomposites with enhanced visible light photocatalytic degradation of ethylene. Appl. Catal. B Environ..

[24] Zeng X.K., Wang Z.Y., Meng N., McCarthy D.T., Deletic A., Pan J.H., Zhang X.W. (2017). Highly dispersed TiO_2_ nanocrystals and carbon dots on reduced graphene oxide: Ternary nanocomposites for accelerated photocatalytic water disinfection. Appl. Catal. B Environ..

[25] Shanmugasundaram A., Baek K.W., Paeng C.G., Li L.L., Cha G., Woo J., Kim D.S., Yim C.Y., Park J.S., Cho J.S. (2025). Dual-sensitized hollow SnO_2_ nanospheres with rGO and Pd for highly sensitive detection of acetone in exhaled breath. Appl. Surf. Sci..

[26] Das D., Nandi P. (2021). Synthesis of CdS/GO modified ZnO heterostructure for visible light dye degradation applications. Appl. Surf. Sci..

[27] Yin Z.Y., Li M., Wang N., Zhou Z.M., Hu Y., Fan D.Y., Pang Y.Y., Liu Y.P., Lu Z.H., Hai J.F. (2023). Deposition of CdS and ZnS directly on rGO via. an emulsion-solvothermal method for excellent photocatalytic activity and stability. Appl. Surf. Sci..

[28] Yang H.Z., Wan Y.Q., Cheng Q.R., Zhou H., Pan Z.Q., Liu Y. (2023). MOF-derived CdS/CoO S-type heterojunctions for improving the efficiency of photocatalytic evolution. Dalton Trans..

[29] Rahman M.S., Suvo M.A.H., Islam M.T., Noor A.R., Yeachin N., Bhuiyan M.A. (2024). Fast and efficient removal of metronidazole from aqueous solution using graphene oxide (GO) supported nitrogen (N) doped zinc oxide (ZnO) nanoparticles. Colloids Surf. A Physicochem. Eng. Asp..

[30] Nguyen C.H., Tran M.L., Tran T.T.V., Juang R.S. (2020). Enhanced removal of various dyes from aqueous solutions by UV and simulated solar photocatalysis over TiO_2_/ZnO/rGO composites. Sep. Purif. Technol..

[31] Feng C., Chen Z.Y., Hou J., Li J.R., Li X.B., Xu L.K., Sun M.X., Zeng R.C. (2018). Effectively enhanced photocatalytic hydrogen production performance of one-pot synthesized MoS_2_ clusters/CdS nanorod heterojunction material under visible light. Chem. Eng. J..

[32] Tang Y.F., Liu X.L., Ma C.C., Zhou M.J., Huo P.W., Yu L.B., Pan J.M., Shi W.D., Yan Y.S. (2015). Enhanced photocatalytic degradation of tetracycline antibiotics by reduced graphene oxide–CdS/ZnS heterostructure photocatalysts. New J. Chem..

[33] Marcano D.C., Kosynkin D.V., Berlin J.M., Sinitskii A., Sun Z.Z., Slesarev A., Alemany L.B., Lu W., Tour J.M. (2010). Improved Synthesis of Graphene Oxide. ACS Nano.

[34] Xiao R., Zhao C.X., Zou Z.Y., Chen Z.P., Tian L., Xu H.T., Tang H., Liu Q.Q., Lin Z.X., Yang X.F. (2020). In situ fabrication of 1D CdS nanorod/2D Ti_3_C_2_ MXene nanosheet Schottky heterojunction toward enhanced photocatalytic hydrogen evolution. Appl. Catal. B Environ..

[35] Wang X.W., Tian H.W., Yang Y., Wang H., Wang S.M., Zheng W.T., Liu Y.C. (2012). Reduced graphene oxide/CdS for efficiently photocatalystic degradation of methylene blue. J. Alloys Compd..

[36] Jiang L.S., Xie Y., He F., Ling Y., Zhao J.S., Ye H., Li S.Q., Wang J.L., Hou Y. (2021). Facile synthesis of GO as middle carrier modified flower-like BiOBr and C_3_N_4_ nanosheets for simultaneous treatment of chromium (VI) and tetracycline. Chin. Chem. Lett..

[37] Zhang Q.Y., Li M., Liu Y.T., Wang X.D., You D.D. (2021). Fabrication of the hierarchical CdS flowers assembled from flakes with (0001) facets exposed as efficient photocatalyst for H_2_ production. Solid State Sci..

[38] Hassan A., Liaquat R., Iqbal N., Ali G., Fan X., Hu Z.L., Anwar M., Ahmad A. (2021). Photo-electrochemical water splitting through graphene-based ZnS composites for H_2_ production. J. Electroanal. Chem..

[39] Senasu T., Chankhanittha T., Hemavibool K., Nanan S. (2021). Visible-light-responsive photocatalyst based on ZnO/CdS nanocomposite for photodegradation of reactive red azo dye and ofloxacin antibiotic. Mater. Sci. Semicond. Process..

[40] Mautschke H.H., Drache F., Senkovska I., Kaskel S., Xamena F.X.L.I. (2018). Catalytic properties of pristine and defect-engineered Zr-MOF-808 metal organic frameworks. Catal. Sci. Technol..

[41] Xu Y.J., Zhuang Y.B., Fu X.Z. (2010). New Insight for Enhanced Photocatalytic Activity of TiO_2_ by Doping Carbon Nanotubes: A Case Study on Degradation of Benzene and Methyl Orange. J. Phys. Chem. C.

[42] Imboon T., Khumphon J., Yotkuna K., Tang I.M., Thongmee S. (2021). Enhancement of photocatalytic by Mn_3_O_4_ spinel ferrite decorated graphene oxide nanocomposites. SN Appl. Sci..

[43] Ghosh S., Basu S., Baskey (Sen) M. (2017). Decorating mechanism of Mn_3_O_4_ nanoparticles on reduced graphene oxide surface through reflux condensation method to improve photocatalytic performance. J. Mater. Sci. Mater. Electron..

[44] Chen Y.L., Chen L., Sung M.Y., Lin J.H., Liu C.J., Kuo C.J., Cho E.C., Lee K.C. (2023). Environment-friendly organic coordination design of Z-scheme heterojunction N-BOB/BiOIO_3_ for efficient LED-light-driven photocatalytic and electrochemical performance. Chemosphere.

[45] Yan J.J., Wang K., Xu H., Qian J., Liu W., Yang X.W., Li H.M. (2013). Visible-light photocatalytic efficiencies and anti-photocorrosion behavior of CdS/graphene nanocomposites: Evaluation using methylene blue degradation. Chin. J. Catal..

[46] Wang H.J., Li J.Z., Wan Y., Nazir A., Song X.H., Huo P.W., Wang H.Q. (2023). Synthesis of AgInS_2_ QDs-MoS_2_/GO composite with enhanced interfacial charge separation for efficient photocatalytic degradation of tetracycline and CO_2_ reduction. J. Alloys Compd..

[47] Kumar R., Sudhaik A., Sonu Raizada P., Nguyen V.H., Le Q.V., Ahamad T., Thakur S., Hussain C.M., Singh P. (2023). Integrating K and P co-doped g-C_3_N_4_ with ZnFe_2_O_4_ and graphene oxide for S-scheme-based enhanced adsorption coupled photocatalytic real wastewater treatment. Chemosphere.

[48] Perumal K., Shanavas S., Ahamad T., Karthigeyan A., Murugakoothan P. (2023). Construction of Ag_2_CO_3_/BiOBr/CdS ternary composite photocatalyst with improved visible-light photocatalytic activity on tetracycline molecule degradation. J. Environ. Sci..

[49] Soori M.M., Ghahramani E., Kazemian H., Al-Musawi T.J., Zarrabi M. (2016). Intercalation of tetracycline in nano sheet layered double hydroxide: An insight into UV/VIS spectra analysis. J. Taiwan Inst. Chem. Eng..

[50] Zhao Y.P., Geng J.J., Wang X.R., Gu X.Y., Gao S.X. (2011). Adsorption of tetracycline onto goethite in the presence of metal cations and humic substances. J. Colloid Interface Sci..

[51] Schlumberger C., Thommes M. (2021). Characterization of Hierarchically Ordered Porous Materials by Physisorption and Mercury Porosimetry—A Tutorial Review. Adv. Mater. Interfaces.

[52] Wang Z.L., Chen Y.F., Zhang L.Y., Cheng B., Yu J.G., Fan J.J. (2020). Step-scheme CdS/TiO_2_ nanocomposite hollow microsphere with enhanced photocatalytic CO_2_ reduction activity. J. Mater. Sci. Technol..

[53] Liao M.J., Zheng Z.L., Jiang H.Y., Ma M.Y., Wang L.M., Wang Y., Zhuang S.T. (2024). MXenes as emerging adsorbents for removal of environmental pollutants. Sci. Total Environ..

[54] Guan Z.L., Li X.M., Wu Y., Chen Z., Huang X.D., Wang D.B., Yang Q., Liu J.L., Tian S.H., Chen X.Y. (2021). AgBr nanoparticles decorated 2D/2D GO/Bi_2_WO_6_ photocatalyst with enhanced photocatalytic performance for the removal of tetracycline hydrochloride. Chem. Eng. J..

[55] Firouzi F., Pirbazari A.E., Saraei F.E.K., Yazdi F.S.T., Esmaeili A., Khodaee Z. (2021). Simultaneous adsorption-photocatalytic degradation of tetracycline by CdS/TiO_2_ nanosheets/graphene nanocomposites: Experimental study and modeling. J. Environ. Chem. Eng..

[56] Zhang M., Dong Y.Y., Yin H.F., Chen X.B. (2022). Construction of CdS/CdSnO_3_ direct Z-scheme heterostructure for efficient tetracycline hydrochloride photodegradation. Mater. Sci. Semicond. Process..

[57] Du C.Y., Zhang Z., Tan S.Y., Yu G.L., Chen H., Zhou L., Yu L., Su Y.H., Zhang Y., Deng F.F. (2021). Construction of Z-scheme g-C_3_N_4_/MnO_2_/GO ternary photocatalyst with enhanced photodegradation ability of tetracycline hydrochloride under visible light radiation. Environ. Res..

[58] Zeng Y., Liu S.T., Zhu G.P., Yang X.T., Yu H.W. (2024). Fe(III)-doped ZnIn_2_S_4_-modified graphene oxide in-situ for the degradation of tetracycline hydrochloride in simulated sunlight. Colloids Surf. A Physicochem. Eng. Asp..

[59] Liu C., Yao A.R., Li W., Xu Q., Yang L., Ge Y.Q., Lan J.W., Lin S.J., Qiu J.H. (2025). Design of GO@TiO_2_ and PDA@CNC decorated gelatin aerogel for efficient adsorption and photocatalytic degradation of organic pollutants. J. Water Process Eng..

[60] Wu F., Zhou F., Zhu Z.Y., Zhan S., He Q.C. (2019). Enhanced photocatalytic activities of Ag_3_PO_4_/GO in tetracycline degradation. Chem. Phys. Lett..

[61] Panbude U., Palwe V., Khairkar R.V., Ravi M., Nagababu P. (2024). Enhanced antibiotic degradation using rGO composites under visible light photocatalysis. J. Environ. Chem. Eng..

[62] Song C.J., Li X.Y., Wang L.P., Shi W.D. (2016). Fabrication, Characterization and Response Surface Method (RSM) Optimization for Tetracycline Photodegration by Bi_3.84_W_0.16_O_6.24_- graphene oxide (BWO-GO). Sci. Rep..

[63] Huo P.W., Zhou M.J., Tang Y.F., Liu X.L., Ma C.C., Yu L.B., Yan Y.S. (2016). Incorporation of N–ZnO/CdS/Graphene oxide composite photocatalyst for enhanced photocatalytic activity under visible light. J. Alloys Compd..

[64] Li K.Q., Jiang Y.Q., Rao W., Li Y.D., Liu X., Zhang J., Xu X.Z., Lin K.F. (2022). Cooperative coupling strategy for constructing 0D/2D carbon nitride composites with strengthened chemical interaction for enhanced photocatalytic applications. Chem. Eng. J..

[65] Siddhardhan E.V., Surender S., Arumanayagam T. (2023). Degradation of tetracycline drug in aquatic environment by visible light active CuS/CdS photocatalyst. Inorg. Chem. Commun..

[66] Cao Y.M., Yue L., Li Z.X., Han Y.H., Lian J., Qin H.P., He S.Y. (2023). Construction of Sn-Bi-MOF/Ti_3_C_2_ Schottky junction for photocatalysis of tetracycline: Performance and degradation mechanism. Appl. Surf. Sci..

[67] Lu J., Sun J.X., Chen X.X., Tian S.H., Chen D.S., He C., Xiong Y. (2019). Efficient mineralization of aqueous antibiotics by simultaneous catalytic ozonation and photocatalysis using MgMnO_3_ as a bifunctional catalyst. Chem. Eng. J..

[68] Ji Y.F., Shi Y.Y., Dong W., Wen X., Jiang M.D., Lu J.H. (2016). Thermo-activated persulfate oxidation system for tetracycline antibiotics degradation in aqueous solution. Chem. Eng. J..

[69] Khan M.H., Bae H., Jung J.Y. (2010). Tetracycline degradation by ozonation in the aqueous phase: Proposed degradation intermediates and pathway. J. Hazard. Mater..

[70] Yang R.X., Zhu Z.J., Hu C.Y., Zhong S., Zhang L.S., Liu B.J., Wang W. (2020). One-step preparation (3D/2D/2D) BiVO_4_/FeVO_4_@rGO heterojunction composite photocatalyst for the removal of tetracycline and hexavalent chromium ions in water. Chem. Eng. J..

[71] Ma Y., Gao N.Y., Li C. (2012). Degradation and Pathway of Tetracycline Hydrochloride in Aqueous Solution by Potassium Ferrate. Environ. Eng. Sci..

[72] Lahootifar Z., Yangjeh A.H., Khataee A. (2023). One-pot decoration of CdS and CdMoO_4_ nanoparticles on g-C_3_N_4_ nanoplates: Boosted photocatalytic degradation of tetracycline. J. Alloys Compd..

[73] Li F., Qiang Z.M., Chen S.Q., Wei J.Y., Li T.H., Zhang D.B. (2020). Synthesis of CdS-loaded (CuC_10_H_26_N_6_)_3_(PW_12_O_40_)_2_ for enhanced photocatalytic degradation of tetracycline under simulated solar light irradiation. RSC Adv..

[74] Zhuang S.T., Zhu X., Wang J.L. (2020). Adsorptive removal of plasticizer (dimethyl phthalate) and antibiotic (sulfamethazine) from municipal wastewater by magnetic carbon nanotubes. J. Mol. Liq..

